# Education

**Published:** 1913-11

**Authors:** 


### Education

A man of mature years and broad scholarship, distinguished as a medical teacher, writer and practitioner, mentioned very casually the fact that some time previously he had read an article containing mathematic formulæ which he could not follow. He arranged with a high school boy to help him. Finding himself stupid, as he expressed it, the lessons were extended over a longer time than had been anticipated, but the formulæ, as well as the general subject in which they were included, were finally mastered. Right here was an illuminating explanation of why this man was what the world calls distinguished. He was willing to learn; he had a mind too grandly simple to be ashamed to learn of one of inferior experience and standing. He was, also, very fond of and proud of his youthful teacher and happy that the latter had succeeded in his later life. Reluctantly, and only in response to cross questioning, he divulged that this success was very largely due to help and influence which the mature student had given to his youthful teacher.

We have unequivocally and consistently advocated formal education as a foundation for license and even matriculation in medical schools. But, in reviewing the lives of the older men in medicine, especially on the sad occasions when it becomes our duty to review those lives in their entirety, it has often been impressed upon us that not merely success in the superficial sense but broad scholarship and generous contribution to the fund of common knowledge have been possible in spite of very meagre formal training in schools.

The man who spends four years in studying medicine and who has all the counts required for entrance, and who has done this work simply because it is required by law, will not wind up his life as well as the man who went to a district school in the winter, who took two courses of medical lectures when he could have got a license under the old law and who studied at odd moments all his life, gaining information from every available source. True success can be won only by the man who is willing and anxious to do more than he is compelled.

We see daily instances of practical failure among the graduates of medical schools that require the most of their matriculants and that have the best equipment for turning out the most finished specimens of physicians. Indeed, the trouble is that they are finished.

The human body can store fat up to double of its own healthy, lean weight. Theoretically, this should suffice for its maintenance for about six months. But the lack of proteid and the acid toxæmia developed from the oxidation of the fat would result fatally in about forty days. Carbohydrate can be stored for about a third of a day's work; proteid for a quarter or a fifth. The fat kind of knowledge can be stored for a long time to come, but the men who depend upon this are unwieldy, sour and lacking in vigor. Real energy production and tissue formation must be acquired daily and used freely.

About the same working strength may be obtained from food at $3.00 a week as from that at ten to fifty cents a portion. It is much the same with education. And, in either case, ingestion does not amount to so much as digestion, absorption and assimilation.

Some of our best elementary education came from Rover, Tiger and the old white horse. We need not be ashamed of it.

The old world civilization exceeded that of aboriginal America largely because the former had the help and training in mastering one's self through mastering an animal due to a relative abundance of domesticable animals, while the latter had practically none but the llama and paco.

The medical profession did not begin with bacteriologists and surgeons. Its foundation was the empiric, the nurse, untrained at that, the quack, the man with the very childlike faith in drugs. Psychotherapy would never have been thought of except at the hint of faith-curists and mesmerists. Spondylotherapy is an eminently respectable subject—the original of it is a word coined by a man so ignorant that he did not even know that pathy means suffering and not treatment. We have been saved from therapeutic nihilism by a critical study of drugs, undoubtedly suggested by the patient though superficial and self-deceptive method of drug "proving." Our knowledge of pyrexia, of blood pressure, of Roentgenology, of bacteriology, of physiologic chemistry and pathologic microscopy, in short, nearly every detail of scientific medicine has been due to hints obtained not, it is true from the ignorant, but from those for the most part ignorant of medical problems. Twenty years ago a large part of what is now termed scientific medicine, lay with young, inexperienced physicians of no professional standing, and it has been developed largely by the boys with whom they talked and who worked up details and took and gave hints. Some of these boys have never even attached themselves to the medical profession. A very considerable part of our practical and theoretical knowledge of bacteriology is due to those whom our fathers in medicines would have despised as horse doctors, and much of our chemistry to agricultural students, despised by their associates in universities and ridiculed by practical farmers.

There is something wrong with ourselves, if we cannot learn something from every person, animal and plant, by careful observation.

